# The Effect of Yttrium on the Solution and Diffusion Behaviors of Helium in Tungsten: First-Principles Simulations

**DOI:** 10.3390/ma15238468

**Published:** 2022-11-28

**Authors:** Mingyu Wu, Yujuan Zhang, Kaikai Qiu, Yaxian Shi, Jingyuan Jin, Changchun Ge

**Affiliations:** Institute of Nuclear Materials, School of Materials Science and Engineering, University of Science and Technology Beijing (USTB), Beijing 100083, China

**Keywords:** tungsten, yttrium, helium, first-principles calculation

## Abstract

We systematically investigated the influence of yttrium (Y) on the evolution behavior of helium (He) in tungsten (W) by first-principles calculations. It is found that the addition of Y reduces the solution energy of He atoms in W. Interestingly, the solution energy of He decreases with decreasing distance between Y and He. The binding energies between Y and He are inversely correlated with the effective charge of He atoms, which can be attributed to the closed shell structure of He. In addition, compared with pure W, the diffusion barrier (0.033 eV) of He with Y is lower, calculated by the climbing-image nudged elastic band (CI-NEB) simulations, reflecting that the existence of Y contributes to the diffusion of He in W. The obtained results provide a theoretical direction for understanding the diffusion of He.

## 1. Introduction

Fusion energy is a kind of environment-friendly energy, which has been considered as an effective way to solve the energy crisis [1]. The development of plasma-facing materials (PFMs) is important in applying nuclear fusion energy [2]. W is one of the candidate materials for the manufacturing of PFMs in future fusion power reactors because W alloys have long service life due to their high melting point, low physical sputtering rate, and having no chemical reaction with hydrogen (H) [3,4]. However, pure W metal exhibits poor fracture toughness and radiation stability and low ductility associated with a high ductile-to-brittle transition temperature [5]. Under a nuclear fusion environment, W PFMs will be irradiated by high-energy neutrons, helium (He), and H isotope plasma, resulting in irradiation embrittlement and surface blistering [6].

The service lifetime of W PFMs can be extended by improving their mechanical stability and irradiation resistance. Doping alloy elements in W has been demonstrated to be a promising method to improve the mechanical properties [7]. Previous works indicate that the improvement of mechanical properties can be achieved by grain refinement [8,9,10]. For example, alloying Y could improve the mechanical properties of W [11,12]. Y plays an important role in affinity oxygen of W, significantly reducing the damage caused by oxygen [13]. It is reported that Y, like lanthanum (La) or cerium (Ce), can help W refine grain size and inhibit recrystallization [14,15,16].

In addition, in fusion devices, PFMs will be bombarded by intense fluxes of high-energy neutrons, H isotopes, and He particles [17,18], leading to retention of H and He atoms inside the PFMs and production of He bubbles and nanostructure, which will seriously reduce the mechanical stability of PFMs, affecting the service life of W-based PFMs [19]. The aggregation of He atoms can form bubbles and substantially deteriorate mechanical material properties [20]. The production of He bubbles will cause the material to become severely embrittled even at very low He concentrations in the structural materials [21]. Therefore, the micro-evolution behavior of these high-energy particles in PFMs is extremely important to study, owing to the intensified structure modification and property degradation [22,23,24]. Zhang et al. investigated the effect of Zr and V in W on He by first-principles calculation and found that the strong attraction between V and He leads to weaker solution energies of He in the region near V. The calculations also revealed that the He atom diffusion to Zr/V has a lower energy barrier than that of He diffusion to W along the optimal diffusion path TIS (tetrahedral interstitial site) -TIS [25]. Yue et al. showed the strong attraction of Re with He clusters in W, the Re addition effectively reducing the diffusion of He clusters. As for the aggregated Re distribution, the He-trapping capability of vacancy is weakened by Re clusters [19]. Based on the above fact, there is a lack of systematic research on the effect of alloying element Y on the solution and diffusion behavior of He in W PFMs.

In this paper, we studied the stability of He and Y atoms in W by using first-principles calculations. The effect of Y on the behavior of He atoms in the W system was studied. The migration and diffusion mechanism of He in W PFMs was achieved by the CI-NEB [26,27] method. Our finding will not only help to understand the interaction mechanism of He and Y in W but also provide a theoretical direction for future fusion reactors.

## 2. Computational Methods

Our studies were obtained with the Vienna Ab-initio Simulation Package (VASP) Ver. 5.4 using the Projector Augmented Wave (PAW) method [28,29,30]. The exchange-correlation potential was described by the Perdew–Burke–Ernzerhof (PBE) functional within the generalized gradient approximation (GGA) [31]. In this paper, the micro-evolution behavior of He has been calculated on the 128-atom (4 × 4 × 4) body-centered cubic (bcc) supercell. The Brillouin-zone integration was performed within Monkhorst–Pack [32] scheme using a 3 × 3 ×3 mesh for the geometry optimizations and 4 × 4 × 4 mesh for electronic calculations in the supercell [33]. A cutoff energy of 350 eV was set for all simulation calculations. The total energy of each system was relaxed until the difference value was smaller than 10^−5^ eV. Each atom was fully relaxed until the force was less than 10^−3^ eV/Å. The fast Fourier transform (FFT) grid of 160 for the VASP parameters NGXF, NGYF, and NGZF was used to calculate the Bader of the system. The diffusion of He in W was obtained using CI-NEB method calculations [19]. It should be noted that the energy convergence criteria used to calculate the diffusion of He in W were less than 10^−5^ eV.

As in our previous study, the Y atoms prefer to occupy the substitute (Sub) site in W [34]. To study the effect of Y atoms on behavior of He in W, when the He atom occupies the Sub site in W system, the solution energy is calculated as described below [35]:

(1) $$E_{\mathrm{Sub},\;W\;-\;Y}^{\mathrm{sol}}=E_{\left(N\;-\;2\right)W,Y+\mathrm{He}}^{T}-E_{\left(N\;-\;2\right)W,Y}^{T}-E_{\mathrm{He}}+E_{\mathrm{Vac}},$$

where $E_{\left(N\;-\;2\right)W,Y+\mathrm{He}}^{T}$ is the total energy of W system containing N−2 W atoms, a single Y and He atoms; $E_{\left(N\;-\;2\right)W,Y}^{T}$ is the total energy of W system containing N−2 W atoms and a single Y atom; $E_{\mathrm{He}}$ is the energy of isolated He atom. $E_{\mathrm{Vac}}$ is the formation energy (3.12 eV) [36] of a vacancy. The solution energy of He atoms at TIS and octahedral interstitial site (OIS) in W-Y system is defined as

(2) $$E_{\mathrm{int},W\;-\;Y}^{\mathrm{sol}}=E_{\left(N\;-\;1\right)W+Y+\mathrm{He}}^{T}-E_{\left(N\;-\;1\right)W+Y}^{T}-E_{\mathrm{He}},$$

where $E_{\left(N\;-\;1\right)W+Y+\mathrm{He}}^{T}$ is the total energy of W system containing N−1 W atoms, a single Y and He atoms. $E_{\left(N\;-1\right)W+Y}^{T}$ is the total energy of W system containing N−1 W atoms and a single Y atom. $E_{\mathrm{He}}$ is the energy of an isolated He atom. Furthermore, the interaction between two defects (Y, He) in W system can be explained by the binding energy ($E_{Y\;-\;\mathrm{He}}^{\mathrm{binding}}$). The $E_{Y\;-\;\mathrm{He}}^{\mathrm{binding}}$ is calculated as follows:

(3) $$E_{Y\;-\;\mathrm{He}}^{\mathrm{binding}}=\left(E_{Y}^{T}+E_{\mathrm{He}}^{T}\right)-E_{Y+\mathrm{He}}^{T}-E_{\mathrm{bulk}}^{T},$$

where $E_{Y+\mathrm{He}}^{T}$ is the total energy of W system containing Y and He; $E_{\mathrm{bulk}}^{T}$ is total energy of pure W system; $E_{Y}^{T}$ and $E_{\mathrm{He}}^{T}$ are total energies of the bulk W with Y and He atoms, respectively. According to the definition of binding energy, negative values indicate repulsion between the defects.

## 3. Results and Discussion

### 3.1. Effect of Y on Solution Energy of He in W

**Figure 1:**
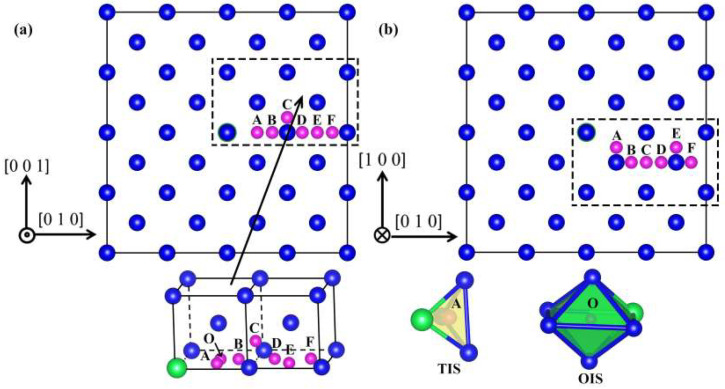
(**a**,**b**) Schematic top and side views of the bulk W-Y system, respectively. Here, A–F and O represent seven different interstitial sites, and the local 3D configuration represents the spatial structure of the interstitial sites (TIS and OIS). Here, the blue, green, and purple balls represent W, Y, and He, respectively.

In the bcc structure W material, all Sub sites are equivalent. Moreover, there are two different interstitial sites, i.e., the TIS and OIS. We use a_0_ to represent the bcc lattice constant. The Sub site has eight nearest neighbors at 0.866 a_0_, which has the largest free volume. The TIS has four nearest neighbors at 0.559 a_0_. The OIS has six nearest neighbors; two of them are located at 0.5 a_0_ and four of them at 0.707 a_0_. We calculated the solution energy of He in the interstitial position of bcc W. As shown in Table 1, the solution energies of He for TIS and OIS are 6.367 eV and 6.510 eV in pure W, respectively. Zhang et al. calculated the solution energy of He for TIS (6.320 eV) and OIS (6.560 eV) [28,37], which agrees well with the results of our calculations. The equilibrium lattice of bcc W was calculated to be 3.170 Å, which is well consistent with the experimental value (3.165 Å) and previous density functional theory results (3.172 Å), indicating that our calculation method is reasonable [38,39]. In addition, we also investigated the effect of Y on the evolution behavior of He atoms in W. Figure 1 is the top and side views of the W-Y system, in which A–F represent TIS and O represents OIS. As shown in Figure 1a, the 3D local view displays the positions of different interstitial and the substitutional sites occupied by the Y atom. The 3D local view in Figure 1b represents the spatial configuration of TIS and OIS.

To investigate the effect of Y on the evolution behavior of He atoms in W, we induce a Y atom in W by doping method. When Y occupies the Sub, the solution energy of He occupied at the nearest neighbor interstitial site of Y is calculated in W. As shown in Table 1, the solution energies of He in the TIS and OIS are 5.263 eV and 5.362 eV, respectively. The most stable configuration for He in interstitial configuration still is the TIS. The addition of Y reduces the solution energies of He atoms in the interstitial sites of the W system. In other words, there is an attractive interaction between Y and He atoms in the W-Y system.

**Figure 2:**
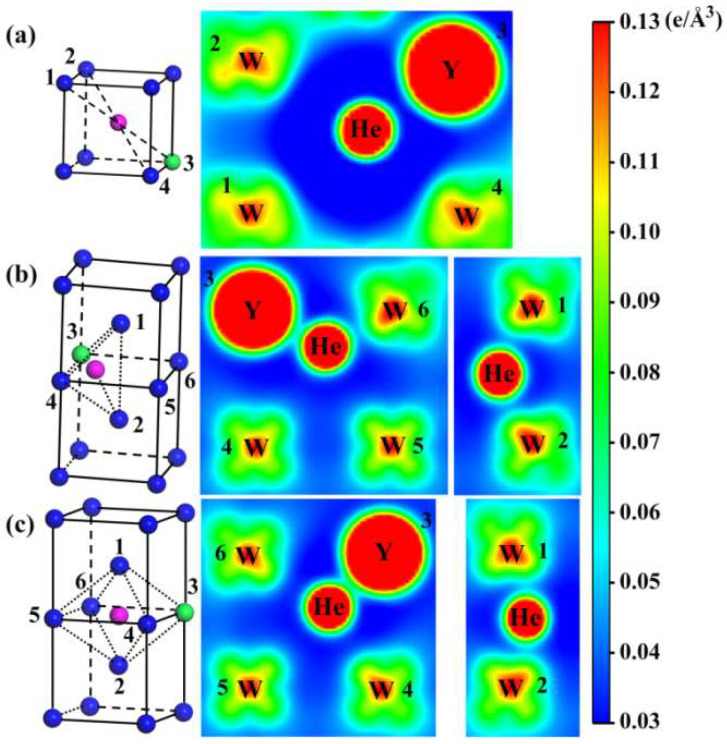
The charge density distribution of He in the bulk W-Y system. (**a**–**c**) correspond to He located at the Sub, TIS, and OIS, respectively. Here, blue, green, and purple balls represent W, Y, and He, respectively.

Our previous work has shown that He prefers to occupy low charge density regions, and the charge density can intuitively explain the relationship between the electronic structure and solution properties of He atoms [25]. Herein, we calculated the charge density and Bader charge of He in the W/W-Y system to analyze the relationship between electronic structure and solution properties. As shown in Figure 2, the charge density distribution of He atoms occupied at different sites in the W-Y system was calculated. Figure 2a–c shows the charge density distributions of W-Y alloys when He atoms occupied Sub, TIS, and OIS sites, respectively. Figure 2a shows the lowest charge density around the He atom at the Sub site, which was favored to be occupied by He and thus reduces the solution energy. Comparing Figure 2b,c, it is clear that the charge density around the TIS occupied by He in the W-Y system is lower than that of the OIS. The lower solution energy of He atoms is at the TIS due to the lower charge density around He. In addition, the solution energies and the corresponding Bader charge of He in the W/W-Y systems are shown in Table 1. Specifically, the Bader charge of He in W is higher than their corresponding occupied sites in the W-Y system. The Bader charges of He atoms at the Sub, TIS, and OIS in the W-Y system are 2.128 e, 2.156 e, and 2.160 e, respectively. The acquired charge of He atoms is inversely correlated with the solution energies. He atoms prefer to keep their electronic structure due to their closed-shell electronic structure. The electron transfer from He atoms can adversely affect its stability.

### 3.2. Interaction between Y and He Atoms

**Figure 3:**
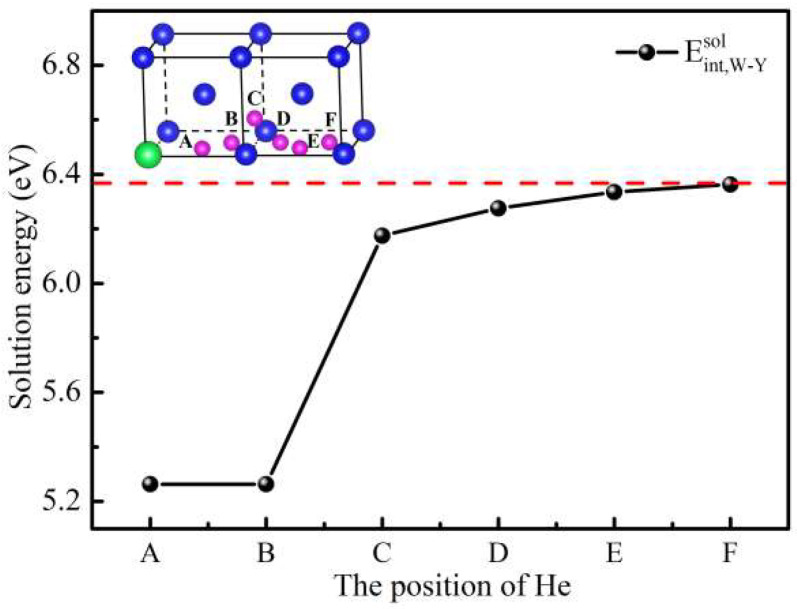
The solution energies of He occupied different TIS in the W-Y system, where the inset 3D model represents the initial positions of the He atoms.

To explore the interaction between Y and He atoms in W, we have calculated the solution energies of He atoms at different TIS (A–F sites) as shown in Figure 3. As displayed in Figure 3, the solution energies of He initially set at the A and B sites are equal. The solution energy increases with distance when He occupies other sites (C–F sites). To deeply reveal the effect of Y on the evolution behavior of He in W, we also calculated the binding energies, Bader charge, and distance between Y and He for different occupied sites as shown in Table 2. It was shown that the solution energy of He atoms at the A site in the W-Y system is the lowest (5.263 eV). The maximum solution energy of He atoms at the F site in the W-Y system is 6.363 eV. To explore the effect of Y on a He solution, the binding energies of Y-He with different distance in the W-Y system were calculated. When He is initially located at the A and B sites in the W-Y system, both binding energies are 1.029 eV. As the distance between Y and He increases, the binding energies gradually decrease until the binding energy (He occupies the F site) is 0.003 eV, indicating that the influence range of Y on the He solution’s characteristics in W is 5.729 Å.

**Figure 4:**
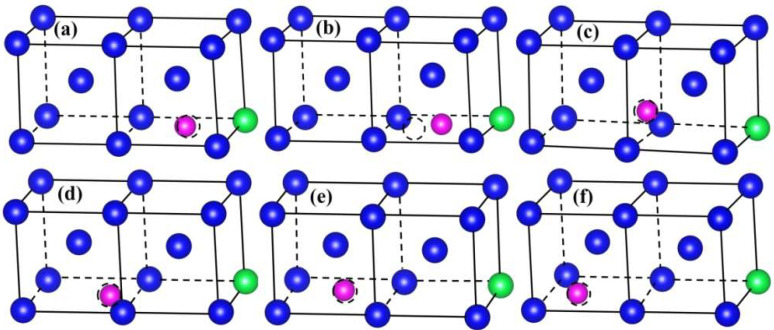
Occupation sites of He in W-Y system after structural optimization. Here, (**a**–**f**) represent He initial at different TIS of A-F, the dotted circles represent the initial occupied sites of He. The blue, green, and purple balls represent W, Y, and He atoms, respectively.

To illustrate the effect of the change of the position of the He atom on the solution energies, we display the configuration of the W-Y system after relaxation. As shown in Figure 4, A–F represent the initial configurations of He atoms at the corresponding A–F sites (TIS), and the dotted circles represent the initial occupied sites of He. From Figure 4a,c–f, it can be known that structural optimization can slightly change the site of TIS (A, C–F site) He in W-Y. It is worth noting that for the initial configuration B in the W-Y system, the final equilibrium configuration after relaxation is the same as that of A, which indicates that He at the B site spontaneously approaches Y. Therefore, there is an attraction between Y and He atoms at the B site in the W-Y system. The final equilibrium configurations of W-Y are consistent with the results of the solution energies of He atoms in the W-Y system.

**Figure 5:**
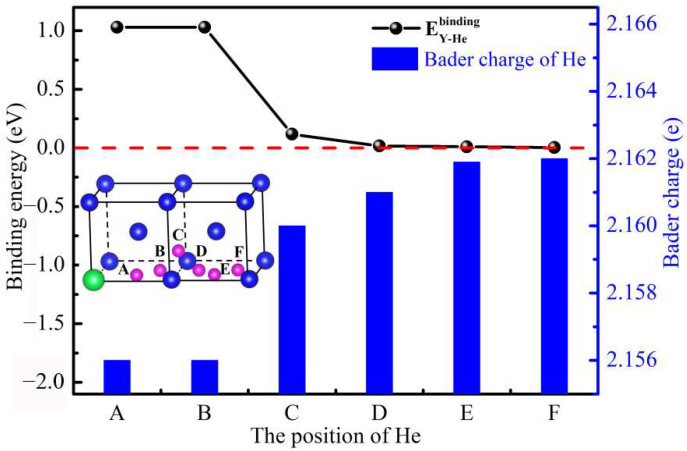
Binding energies and Bader charge of He atoms in the bulk W-Y system, with atomic model schematics representing He at different TIS (A–F sites).

Results reveal the relationship between the binding energies of Y-He and the Bader charge as shown in Figure 5. The binding energies of Y-He in the W-Y system decrease with the increase of the distance between Y and He. Specifically, when He is initially set at the A and B sites in the W-Y system, the Bader charge of He after relaxation is the same. We find that the binding energies of Y-He are inversely proportional to the Bader charge of He atoms. The main factor is that He is a closed shell structure, and the atoms tend to maintain the inherent electronic structure. Therefore, He tends to occupy regions of low charge density. The addition of Y in the bulk W can provide a low charge density region (A site) for He atoms, which is the main contributor for the large binding energy of Y-He.

**Figure 6:**
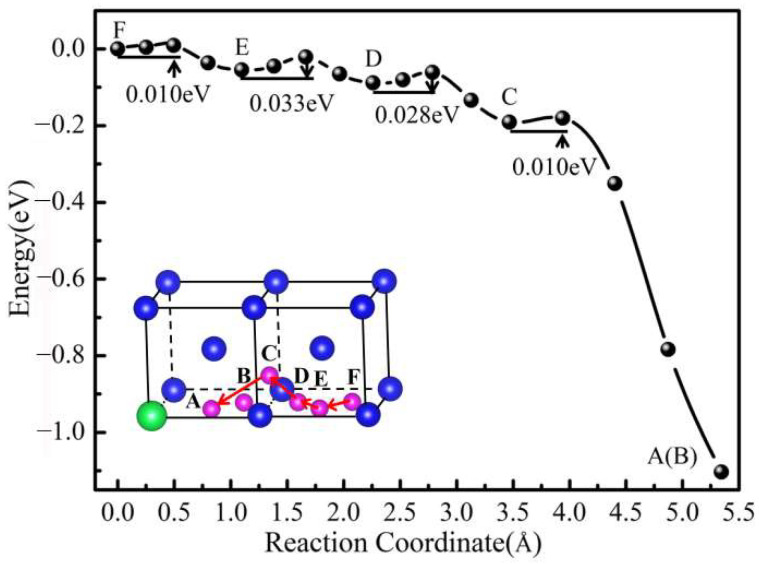
Diffusion barrier for He moving from a far TIS (F site) to TIS (A site) near Y in W-Y system, and the atomic model diagram represents the migration path of He. The blue, green, and purple balls represent W, Y, and He atoms, respectively.

The migration of He plays an acritical role on the microstructure evolution of W after He ions irradiation [41]. Here, we studied the influence of Y on the diffusion and migration behavior of He in the W-Y system. Subsequently, we further studied the diffusion kinetically of He in the W-Y system by the CI-NEB method. As shown in Figure 6, we investigated the He migration barrier by possible paths of He diffusion from the farthest TIS (F site) to the TIS (A site) near Y. The migration barriers and corresponding diffusion paths for He diffusing to a Y atom are studied and displayed in Figure 6. As illustrated in Figure 6, the migration barriers of F → E, E → D, D → C, and C → A are 0.010 eV, 0.033 eV, 0.028 eV, and 0.010 eV, respectively. The path with the largest migration barrier for He in the W-Y system is E → D. The atomic structure analysis showed that the equilibrium configuration of the initial site (B site) of He is the same as that of A site, indicating that there is no migration barrier for the B → A migration path of He. Moreover, the calculated migration barriers (0.057–0.060 eV) [40,42] of the He along the optimal diffusion path TIS → TIS in pure W are higher than the migration barriers (0.033 eV) of He diffusing to the Y atom in W-Y systems. In other words, the diffusion barriers of He from the far occupation sites to the region near Y are smaller than the diffusion of He in pure W. We suppose that the main factor for the lower migration barrier is the stronger attraction between Y and He atoms.

## 4. Conclusions

We have systematically investigated the effect of Y on the evolution behavior of He atoms in W by first-principles calculations. The solution energies of He at the Sub, TIS, and OIS in the W-Y system are 4.670 eV, 5.263 eV, and 5.362 eV, respectively. More importantly, the addition of Y reduces the solution energy of He atoms in W. The solution energies of He increase as the distance from Y to He increases in the W-Y system. The electronic structure analysis indicates that the charge transfer of He in the W-Y system is smaller than the charge change of He atoms at the corresponding sites in pure W. Interestingly, the binding energies between Y and He are inversely correlated with the Bader of He atoms. By analyzing the relationship between binding energies and distance, it was found that the influence range of Y on the He solution’s characteristics in W is 5.729 Å. Finally, the diffusion by the CI-NEB study shows that the migration barrier (0.033 eV) of He near the Y is lower due to the stronger interaction between W and He atoms in the W-Y system.

## Figures and Tables

**Table 1:** Solution energy and Bader charge of He atoms at different occupied sites of W/W-Y systems.

System		Sub	TIS	OIS	
Bulk W	Solution energy (eV)	4.809	6.367	6.510	Present
		4.770 ^a^	6.32 ^b^	6.56 ^b^	Reference
		0.039	0.047	0.050	Error intervals
	Bader charge (e)	2.139	2.159	2.165	Present
Bulk W-Y	Solution energy (eV)	4.670	5.263	5.362	Present
	Bader charge (e)	2.128	2.156	2.160	Present

^a^ Reference [40]. ^b^ Reference [27].

**Table 2:** The solution energy, binding energy, Bader charge, and distance from He to Y in the equilibrium W-Y system.

Site	A	B	C	D	E	F
Solution energy (eV)	5.263	5.263	6.175	6.275	6.335	6.363
Binding energy (eV)	1.029	1.029	0.117	0.017	0.008	0.003
Bader charge (e)	2.156	2.156	2.160	2.161	2.171	2.173
Distance (Å)	1.926	1.926	3.507	4.241	4.860	5.729

## Data Availability

Data are available on request.
